# Ethanol extract of *Polygonatum cyrtonema* Hua mitigates non-alcoholic steatohepatitis in mice

**DOI:** 10.3389/fphar.2024.1487738

**Published:** 2025-01-30

**Authors:** Dongliang Chen, Yue Shen, Fang Huang, Bo Huang, Shangfu Xu, Lisheng Li, Jie Liu, Zheng Li, Xia Li

**Affiliations:** ^1^ Key Laboratory of Basic Pharmacology of Ministry of Education and Joint International Research Laboratory of Ethnomedicine of Ministry of Education, Zunyi Medical University, Zunyi, China; ^2^ Department of Pharmacy, Bijie City Qixingguan District Hospital of Traditional Chinese Medicine, Bijie, Guizhou, China; ^3^ Key Laboratory of Cell Engineering of Guizhou Province, Affiliated Hospital of Zunyi Medical University, Zunyi, China; ^4^ Department of Pharmacology, Key Laboratory of Basic Pharmacology of Guizhou Province and School of Pharmacy, Zunyi Medical University, Zunyi, Guizhou, China

**Keywords:** *Polygonatum cyrtonema* Hua, NASH, AMPK, SIRT1, NF-κ B, PPAR-α, RNA-Seq

## Abstract

**Background:**

*Polygonum cyrtonema* Hua is a kind of traditional Chinese botanic drug. Modern pharmacological research has confirmed that *Polygonum cyrtonema* Hua is able to alleviate nonalcoholic fatty liver disease, but the precise mechanism requires further investigation. This study investigated the protective effects and underlying mechanisms of *Polygonatum cyrtonema* ethanol extract (PCE) against Non-alcoholic steatohepatitis (NASH) in mice.

**Methods:**

UHPLC-MS/MS was utilized to analyze the metabolites of PCE. The NASH mouse model was establishment in C57BL/6J mice via high-fat diet (HFD) feeding for 12 weeks, and from the 9th week, mice were gavaged with PCE (100, 300, and 900 mg/kg/day), simvastatin (4 mg/kg) or saline. One hand, liver injury was assessed by serum enzymes, biochemistry, and histopathology; On the other hand, RNA-seq, qPCR, and Western blot were employed to investigate the related molecular mechanisms.

**Results:**

211 metabolites were identified through UHPLC-MS/MS analysis. PCE ameliorated HFD induced liver injury and improved hepatocellular degeneration and steatosis in a dose-dependent way. PCE restored the expression of AMPK, SIRT1, SREBP1 and PPAR-α both in mRNA and protein levels. RNAseq identified unique gene expression profiles in response to high-fat diet (HFD) compared to the PCE treatments. HFD-induced DEGs were attenuated or abolished following PCE treatments. Ingenuity pathway analysis of RNA-seq data revealed key canonical pathways and upstream molecules regulated by PCE.

**Conclusion:**

Our findings confirm the ability of PCE in alleviating NASH and underscores AMPK/SIRT1 pathway as a potential theraputic target for NASH treatment.

## Introduction

Non-alcoholic fatty liver disease (NAFLD) is a metabolic syndrome marked by excessive fat accumulation in liver cells. NAFLD may initially present as simple steatosis, namely, Non-alcohol-associated fatty liver (NAFL), but can progressively develop into non-alcoholic steatohepatitis (NASH), liver fibrosis, cirrhosis, and potentially liver cancer (Friedman et al., 2018). NASH, an advanced form of NAFL, is more susceptible to develop into fibrosis, cirrhosis and hepatocellular carcinoma and is associated with need for liver transplantation (Geier et al., 2021). Nowadays, approximately 30% of the global population suffers from NAFLD (Younossi et al., 2023). Notably, about 20% of patients with NAFL progress to NASH, and over 40% of patients with NASH progress to fibrosis (Sheka et al., 2020). Therefore, the treatment of NASH can effectively prevent the progression of NAFLD.

Despite advancements in pathological research of NASH, its clinical treatment mainly relies on lifestyle changes at present. Although Rezdiffra was approved by the U.S. Food and Drug Administration in March 2024 to treat patients with NASH, its long-term surveillance is essential to identify potential risks related to thyroid, gonadal, or bone diseases (Petta et al., 2024). Meanwhile, as the global burden of NASH continues to grow, effective NAFLD treatment drugs are still lacking in clinical practice.

Traditional Chinese medicine employs a multi-target, multi-pathway strategy for disease treatment and has been widely utilized in the prevention and management of disease. *Polygonatum cyrtonema* Hua is a perennial plant with a long history for both medicinal and edible use. Containing a variety of bioactive metabolites, *Polygonatum cyrtonema* Hua has shown therapeutic effects in osteoporotic, fatigue and respiratory problems (Cui et al., 2018). In addition, the role of *Polygonatum cyrtonema* Hua in NAFLD has been discovered recently (Liu et al., 2022), however the specific mechanism remains nebulous.

AMP-activated kinase (AMPK) is a vital metabolic sensor in mammals and is activated when ATP levels decreased (Wang Q et al., 2018). The silent information regulator sirtuin 1 (SIRT1) protein is a highly conserved NAD^+^-dependent deacetylase, and regulates fundamental biological functions such as genomic stability, energy metabolism, inflammation and tumorigenesis (You and Liang, 2023). It has been confirmed that activation of AMPK increases intracellular NAD^+^ concentration and triggers activation of SIRT1 (Han et al., 2016). Meanwhile the inhibition of SIRT1 activity induced decreased AMPK activation (Zhang et al., 2024). AMPK and SIRT1 are important players in the coordinating network of cell homeostasis and are largely interdependent to function optimally (Wang L. et al., 2018). The current work was aim to determined the effect of *Polygonatum cyrtonema* Hua in alleviating NASH and whether its effect is related to the activation of AMPK/SIRT1 pathway. Additionally, the metabolites in PCE were systematically analyzed by UHPLC-MS/MS and the gene expression was comprehensively analyzed by RNA-seq.

## Materials and methods

### Preparation of PCE

Dry powder of *Polygonatum cyrtonema* Hua purchased from Guizhou Hengfenghao Agricultural Development Co., Ltd. (Guizhou, China) was soaked in 75% ethanol and extracted by ultrasonic at 40°C. Then the extraction liquid was filtered and concentrated by rotary evaporator. The residue was dried and was extracted again by the method above to obtained the secondary residue and concentrated solution. The secondary residue was soaked in distilled water and then concentrated at 60°C, combined the above concentrated solution and freeze dried the extraction. The PCE yield was around 40% and contained amino acids, flavonoids, polysaccharides, saponins, alkaloids, lectin, and others using UHPLC-ESI-MS/MS analysis by Sanshu Biotechnology (Shanghai, China).

### UHPLC-MS/MS analysis PCE chemical constituents

Take an appropriate volume of sample (0.5–1 mL), add 1 mL water: acetonitrile: isopropanol (1:1:1, v/v) solution for metabolite extraction. The solution underwent 30 min of sonication at 4°C. Following centrifugation (20 min, 12,000 rpm, 4°C), the supernatant was transferred to clean microtubes. Until analysis, samples were freeze-dried and stored at −20°C. For UHPLC-ESI-MS/MS analysis, samples were dissolved in 200 μL of 30% ACN (v/v) and transferred to insert-equipped vials. The analysis was conducted using Thermo Xcalibur 4.0.

### Experimental animals

Male C57BL/6J mice, weighing between 18 and 22 g and of SPF grade, were obtained from Specific Biotechnology Co. Ltd. (Certificate No: SCXK 2019-0010, Beijing, China). All mice were housed in an SPF-grade facility with unrestricted access to food and water. The environment was maintained at 25°C ± 2°C and 50% ± 5% humidity. A 12-h light/dark cycle was maintained. All procedures received approval from the Experimental Animal Ethics Committee of Zunyi Medical University (No. 2020-2-060, Zunyi Medical Lun Audit).

### Animals treatments

Following a 7 days of adaptive feeding, mice were randomly assigned to six groups (n = 6): control, HFD model, HFD + PCE low-dose (100 mg/kg), HFD + PCE medium-dose (300 mg/kg), HFD + PCE high-dose (900 mg/kg), and positive control (simvastatin, 4 mg/kg). Mice in normal control group were fed on a normal chow diet, meanwhile, mice in other groups were fed on a high-fat diet for 12 weeks. From the ninth week, mice in PCE and simvastatin groups were correspondingly administered low-dose PCE, medium-dose PCE, high-dose PCE, or simvastatin via gavage for 4 weeks, mice in control group and HFD group were administered equal volumes of saline. After 12 weeks of high-fat diet feeding, all mice were anesthetized.

### Chemicals and reagents

The high-fat diet was obtained from Ready Dietech in Shenzhen, China. Assay kits for alanine transaminase (ALT, C009-2-1), aspartate transaminase (AST, C010-2-1), triacylglycerol (TG, A110-1), total cholesterol (TC, A111-1), high-density lipoprotein cholesterol (HDL-C, A112-1), and low-density lipoprotein cholesterol (LDL-C, A113-1) were procured from Nanjing Jiancheng Bioengineering Institute (Nanjing, China). All HPLC-grade solvents were obtained from ANPEL Laboratory Technologies (Shanghai, China).

### Biochemical parameter detection

Blood samples were centrifuged to collect serum supernatants. Serum biochemical indicators were tested by commercial kits from Nanjing Jianjian Bioengineering Institute, following the manufacturer’s instructions.

### H&E staining

The same part of mouse liver were fixed in 10% neutral buffered formalin for 48 h and then embedded in paraffin and sliced into 5 μm-thick slides. After dewaxing and dehydration, slides were stained with hematoxylin. Then after differentiation and bluing, slides were stain with eosin. Using a series of gradient alcohol solutions for dehydration, slides were sealed with neutral resin. The pathological changes of mouse liver tissue were observed under an optical microscope.

### Oil red O staining

The frozen liver tissue slides were rewarmed at room temperature, and washed with distilled water firstly to wash off the embedding agent and then washed with 60% isopropyl alcohol for 2 min. All sections are stained with staining solution for 25 min 60% isopropyl alcohol was used to adjust the color. Slides were restained with hematoxylin for 10–20 s and returned to blue with PBS. Glycerol gelatin was used to seal the slides. After drying at room temperature, slides were observed under the light microscope.

### RNA isolation and sequencing

Total RNA was extracted using Trizol reagent (Takara, Japan). Fragmented mRNA samples were reverse transcribed into cDNA using random Oligo dT primers and M-MuLV. Double-stranded cDNA was synthesized using the generated first-strand cDNA, RNase H enzyme, DNA polymerase I, and dNTPs. Purified double-stranded cDNA underwent end repair, A-tailing, and adaptor addition. Screen 250-300bp cDNA using AMPure XP beads, followed by PCR amplification and subsequent purification with AMPure XP beads. The library concentration was measured using Qubit 2.0 and diluted to 1.5 ng/μL. The Agilent 2100 detected the inserted library (420–650 bp, tail <1 Kb), and qPCR confirmed an effective library concentration >3 nM. RNA sequencing was performed using the Illumina NovaSeq PE150 platform. Chongqing Knorigene Technologies (Chongqing, China) conducted the sequencing and bioinformatics analyses following successful library construction. DESeq2 was employed for RNA-seq data analysis, with comparisons made to the Control group. Differentially expressed genes (DEGs) were determined using a significance threshold of *p* < 0.05.

### Reverse transcription-quantitative polymerase chain reaction

After quantifying at 260/280 nm, the total RNA were reverse transcribed by Prime Script TM RT Kit (Takara Biotechnology Co., Ltd., China). Primers were designed using Primer 3 and synthesized by Sangon Biotech and were listed in Table 1. The PCR reaction mixture consisted of 7.5 μL SYBR Super Mix, 0.5 μL of each 10 μM primer, 3 μL cDNA, and 3.5 μL DEPC water, making a total volume of 15 μL. The cycling conditions were set at 95°C for 10 min (1 cycle), 95°C for 10 s and 60°C for 1 min (40 cycles), followed by 95°C for 1 min, 55°C for 1 min, and 55°C for 10 s (80 cycles) for the melting curve. Gene expression levels were measured using quantification cycle (Cq) values. Glyceraldehyde-3-phosphate dehydrogenase (GAPDH) gene expression levels served as the internal control. Each group was normalized to the Control group.

**TABLE 1:** Main experimental primers.

Gene	Forward primer	Reverse primer
SREBP-1c	GCT​AGC​TAG​ATG​ACC​CTG​CAC	GCA​GCA​GCA​AGA​TTT​GCC​TA
PPAR-α	ACT​GGT​AGT​CTG​CAA​AAC​CAA​A	AGA​GCC​CCA​TCT​GTC​CTC​TC
MTTP	ATG​ATC​CTC​TTG​GCA​GTG​CTT	TGA​GAG​GCC​AGT​TGT​GTG​AC
TNF-α	GGC​CTC​CCT​CTC​ATC​AGT​TC	CAC​TTG​GTG​GTT​TGC​TAC​GA
GAPDH	TGT​GTC​CGT​CGT​GGA​TCT​GA	CCT​GCT​TCA​CCA​CCT​TCT​TGA
IL-1β	TGT​GAA​ATG​CCA​CCT​TTT​GA	GGT​CAA​AGG​TTT​GGA​AGC​AG
ApoB	TGAATGCACGGGCAATGA	GGC​ATT​ACT​TGT​TCC​ATG​GTT​CT
SIRT1	GTT​GTG​TGC​CTT​CGT​TTT​TGG​A	AGG​CCG​GTT​TGG​CTT​ATA​CA
AMPK	ATG​GCG​CGA​CCC​GGG​CTT​CTT​CT-3	TAC​CGC​GCT​GGG​CCC​GAA​GAA​GA

### Western blot analysis

Livers were homogenized with RIPA buffer containing PMSF and phosphatase inhibitor. The total protein concentrations were determined by BCA protein assay kit (General Biotech. Co., Shanghai, China). 30 μg of protein was separated using SDS-PAGE and transferred to a PVDF membrane for immunoblotting. After blocking with 5% skimmed milk, membranes were incubated with primary antibodies MTTP (Santa, #C2218), SREBP-1c (Abbkin, #ABP53239), PPAR-α (Abbkin, #ABP55667), ApoB (Proteintech, #20578-1-AP), SIRT1 (Abcam, #ab110304), P-AMPK (CST, #5759), AMPK (CST, #2603), P-ACC (CST, #50081), and ACC (CST, #3662) overnight at 4°C.Membranes were incubated with secondary antibody at room temperature for 1 h, followed by washing. Chemiluminescence ECL developed the color, and protein bands were quantified using the ChemiDoc MP imaging system (Bio-Rad, United States).

### Ingenuity pathway analysis

Canonical pathway and upstream regulator analyses were performed using the Ingenuity pathway analysis (IPA) server (Qiagen, Redwood City, CA). The IPA software determines significance using a right-tailed Fisher’s Exact test, with the P-value indicating the probability of overlap between the treatment groups and the IPA pathway gene list. Additionally, upstream analysis employed differentially expressed genes to identify regulatory factors, with variations across treatment groups assessed using the Z-score.

### Statistical analysis

The experimental data were statistically analyzed by GraphPad Prism 8.0 software. Quantitative data were presented as mean ± SEM. A t-test compared two groups, while one-way ANOVA with LSD *post hoc* test was used for multiple group comparisons. *p* < 0.05 signifies statistical significance.

## Results

### Identification of the constituents of PCE

**FIGURE 1:**
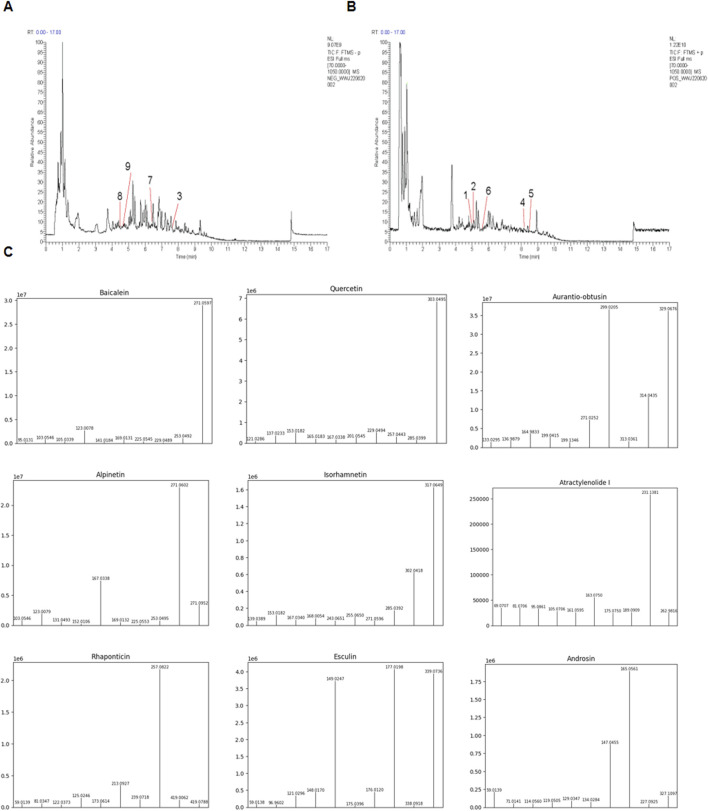
Total ion chromatograms of PCE. **(A)** Representative chromatography of PCE using the negative mode; **(B)** Representative chromatography of PCE using the positive mode; **(C)** UHPLC-ESI-MS/MS spectra of the identified 9 lipid - lowering metabolites from PCE.

UHPLC-ESI-MS/MS analysis was adopted to determine metabolites in PCE. The mass spectral total ion chromatogram of PCE was analyzed by UHPLC-ESI-MS/MS in positive and negative ion modes, as shown in Figures 1A, B. By comparing it with the database, a total of 211 metabolites were identified in PCE and 9 of which were reported to exhibit lipid-lowering effect. These 9 metabolites include baicalin (Guo et al., 2023), quercetin (Yang H. et al., 2019; Yang J. et al., 2019), and aurantio-obtusin (Li HY et al., 2023), atractylenolide (Li et al., 2022), alpinetin (Zhou et al., 2018), isorhamnetin (Ganbold et al., 2019), rhaponticin (Chen et al., 2009), esculin (Yang et al., 2021), and androsin (Singh et al., 2024). The main information of the 9 lipid-lowering metabolites is shown in Table 2 and their MS/MS fragment ions spectrum are displayed in Figure 1C.

**TABLE 2:** Identification of main metabolites in PCE by UHPLC-ESI-MS/MS analysis.

	Name	Formula	Mode	Remain time (min)
1	Baicalein	C_15_H_10_O_5_	POS	4.98
2	Quercetin	C_15_H_10_O_7_	POS	4.99
3	Aurantio-obtusin	C_17_H_14_O_7_	NEG	7.38
4	Atractylenolide	C_15_H_20_O_3_	POS	8.20
5	Alpinetin	C_16_H_14_O_4_	POS	8.47
6	Isorhamnetin	C_16_H_12_O_7_	POS	5.50
7	Rhaponticin	C_21_H_24_O_9_	NEG	6.37
8	Esculin	C_15_H_16_O_9_	NEG	4.39
9	Androsin	C_15_H_20_O_8_	NEG	4.24

### Protective effect of PCE on HFD-Induced nonalcoholic fatty liver in mice

**FIGURE 2:**
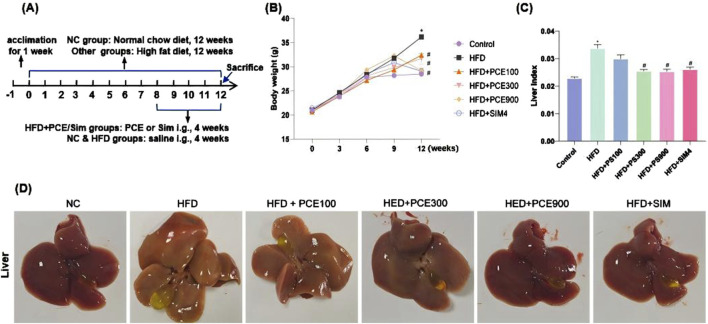
PCE alleviated the HFD-induced increases of body weight and liver index in mice. **(A)** The time modeling process of HFD-induced NASH mouse model, **(B)** Representative picture of liver morphology; **(C)** Weekly weight measurement curve; **(D)** Liver index (liver/body weight, g/g). Data are shown as mean ± SEM (n = 6).*p < 0.05 compared to Control; #*p* < 0.05 compared to Model.

For establishing a NASH mouse model, C57BL/6 mice were fed with HFD for 12 weeks, and the detailed experimental timeline is shown in Figure 2A. NAFLD begins with hepatic fat accumulation, and after 12 weeks HFD feeding, mice exhibited yellowish livers due to lipid deposition. In addition, the livers’ volume were obviously increased and accompanied by significant enlarger gallbladders (Figure 2A).

In PCE groups, mice were gavaged different dose of PCE continuously for 4 weeks, which reversed HFD-induced increasing weight gain and liver index as shown in Figures 2B, C. Additionally, the color and volume of mice livers gradually approached normal in a dose-dependent manner (Figure 2D). Simvastatin is effective in reducing blood lipid levels and could be used as an appropriate positive control group for NAFLD (Yang et al., 2024). Our results indicated that simvastatin effectively reduced the increase of body weight and liver index in mice induced by HFD feeding. High doses of PCE and simvastatin showed almost the same improvement in lowering mice body weight and liver index.

### PCE ameliorated HFD-induced histological changes and lipid metabolism disorder

**FIGURE 3:**
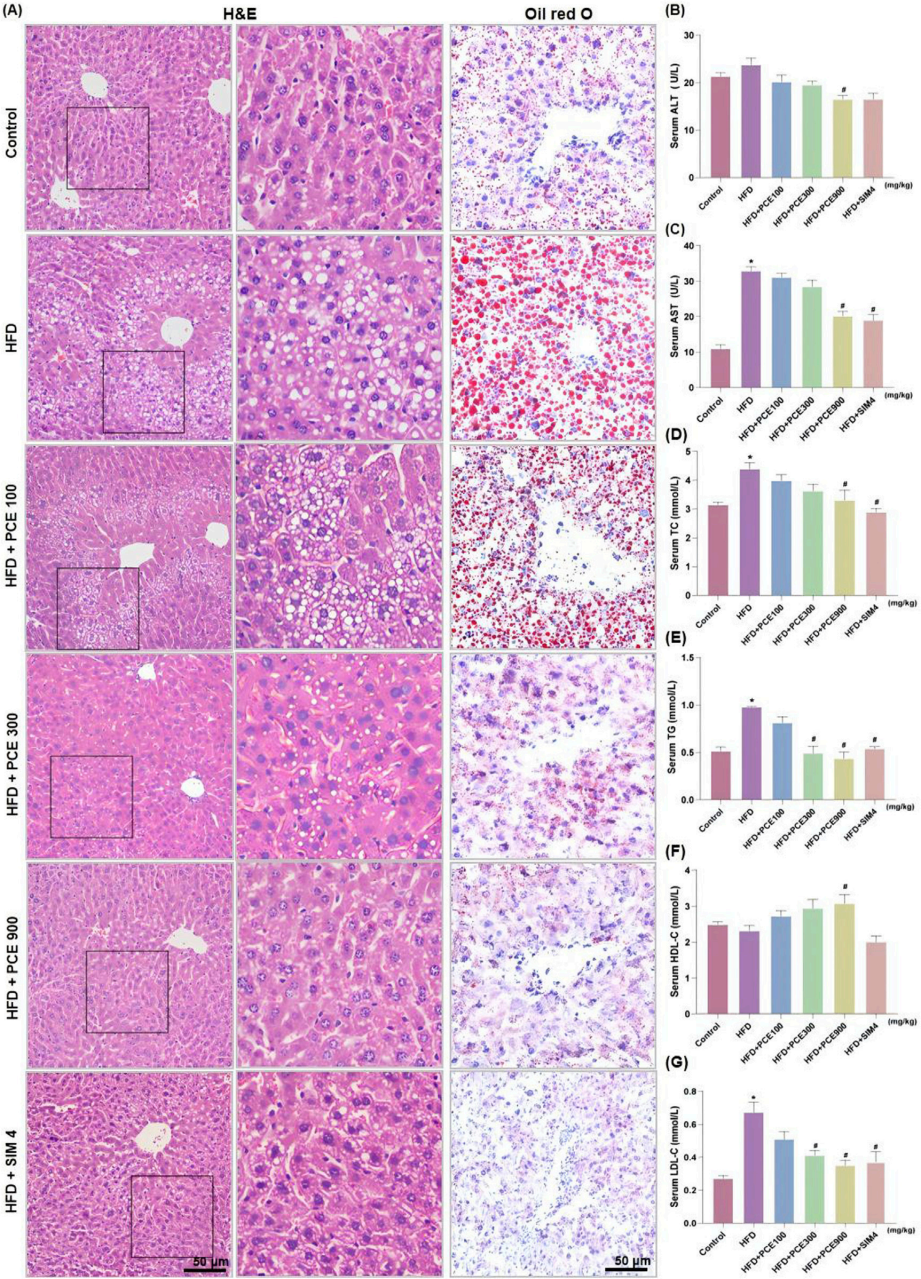
PCE treatment relieved HFD-induced liver injury and hepatosteatosis. **(A)** Representative H&E staining images and Oil red O staining images (×200, the middle panel is partly magnification of H&E staining images); **(B)** Serum ALT; **(C)** Serum AST; **(D)** Serun TC; **(E)** Serum TG; **(F)** Serum Serum high-density lipoprotein (HDL-C); **(G)** Serum low density lipoprotein (LDL-C). Data are presented as mean ± SEM (n = 6). ^*^ *p* < 0.05 compared to Control; ^#^P< 0.05 compared to HFD group.

H&E staining and Oil red O staining were adopted to observe the effect of PCE on histological changes and hepatic steatosis. Liver tissue of HFD mice exhibited obvious wide distribution of fatty vacuolation in H&E staining and red lipid droplets in Oil red O staining. Meanwhile, hepatocytic ballooning, a unique form of hepatocyte injury emerged in liver tissue of HFD group. Similarly, PCE treatment significantly ameliorated these histopathological changes in a dose-dependent manner (Figure 3A). The relative area of fat vacuoles in H&E staining and relative area of lipid droplets in Oil red O staining showed the same trend with staining results (Supplementary Figure S1).

To quantitative analysis the impact of PCE on alleviating HFD-induced liver injury, the levels of alanine aminotransferase (ALT) and aspartate aminotransferase (AST) were measured. HFD feeding significantly elevated serum levels of ALT and AST. Consistantly, both the high dose of PCE and simvastatin, effectively reduced the serum levels of AST and ALT (Figures 3B, C). PCE’s lipid-lowering effect was further certificated by the reduced serum triglyceride (TG) and total cholestrol (TC) levels (Figures 3D, E). HFD induced NAFLD is often accompanied by cholesterol metabolism disorders (Li et al., 2021). Fortunately, PCE showed the ability to restore the balance of cholesterol metabolism (Figures 3F, G), and surprisingly, PCE upregulated plasma concentration of HDL-C (Figure 3G), which is is negatively associated with the development of cardiovascular disease (Deprince et al., 2020). These results highlight the remarkable potential of PCE in regulating lipid metabolism.

### PCE reversed HFD-induced hepatic steatosis through regulating lipid synthesis and fatty acid oxidation

**FIGURE 4:**
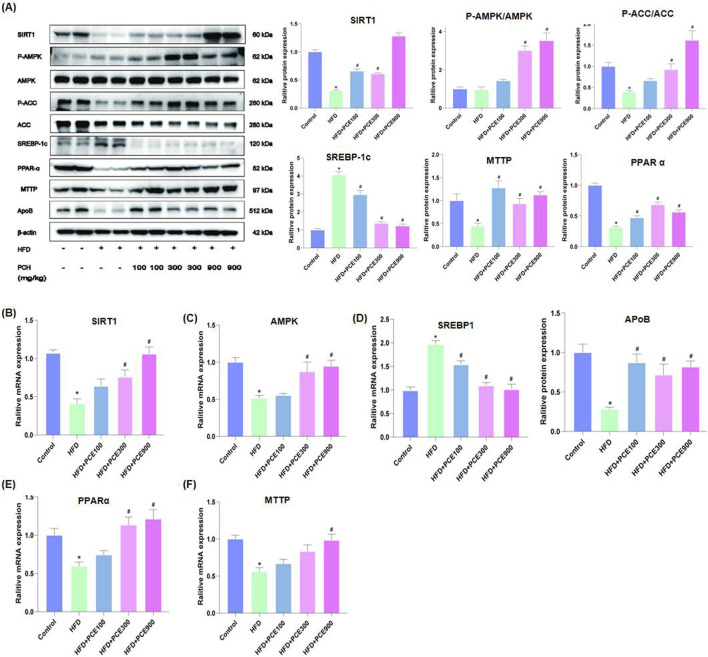
PCE treatment activated the AMPK/SITR1 signalling pathway. **(A)** Representative Western bolt results of SIRT1, AMPK, ACC, SREBP-1c, PPAR-α, MTTP, and ApoB and quantification results. **(B)** mRNA relative expression level of SIRT1; **(C)** mRNA relative expression level of AMPK; **(D)** mRNA relative expression level of SREBP1; **(E)** The mRNA relative expression level of PPAR-α; **(F)** The mRNA relative expression level of MTTP. Data are presented as mean ± SEM (n = 6). ^*^ *p* < 0.05 compared to Control; ^#^ *p* < 0.05 compared to HFD group.

Given the excellent performance of PCE in reversing HFD-induced hepatic steatosis, we further determined the expression level of AMPK/SIRT1 in the liver tissue of each group by Western blot and qPCR. Activity of AMPK reduced by obesity, diabetes as well as NAFLD (Smith et al., 2016), which was confirmed by our data again. HFD feeding decreased the ratio of phosphorylated AMPK to total AMPK. PCE administration restore the expression of AMPK and phosphorylated AMPK. Similarly, numerous studies have point out that SIRT1 harnesses multiple pathways to hinder NAFLD (Tian et al., 2024). Consistent with this, HFD-induced decline in SIRT1 expression was restored after PCE administration (Figures 4A–C).

Enhanced *de novo* lipogenesis in hepatocytes plays an important role in the process of NAFLD. Sterol regulatory element-binding proteins (SREBPs) are key transcriptional factors for genes in the *de novo* lipogenesis pathway, such as acetyl CoA carboxylase (ACC), which is responsible for catalyzing the rate-limiting step of fatty acid synthesis (Zeng et al., 2022). Additionally, AMPK is involved in mitochondrial fatty acid β oxidation via activating peroxisome proliferation-activated receptor α (PPAR-α). Through regulating SREBP1 pathway and PPAR-α pathway, AMPK takes parts in the process of lipid metabolism (Li et al., 2011). Therefore, to further confirm the effects of PCE on activating AMPK/SIRT1, we determined the expression of SREBP1, ACC and PPAR-α by Western blot and qPCR. As show in Figures 4A–F, HFD-induced protein or mRNA level changes of these molecular were restored by PCE.

Dyslipidemia is often observed in NAFLD patients (Deprince et al., 2020), as well as in our mouse model. Therefore we also evaluated the expression of apolipoprotein B (ApoB) and microsomal triglyceride transfer protein (MTTP), which are crucial for very low-density lipoprotein (VLDL) secretion and lipid homeostasis in the liver (Peng et al., 2021). After a high-fat diet feeding, the amount of apoB and MTTP in the liver decreased, and which were reversed by PCE treatment.

### PCE reversed HFD-induced hepatic inflammatory response

**FIGURE 5:**
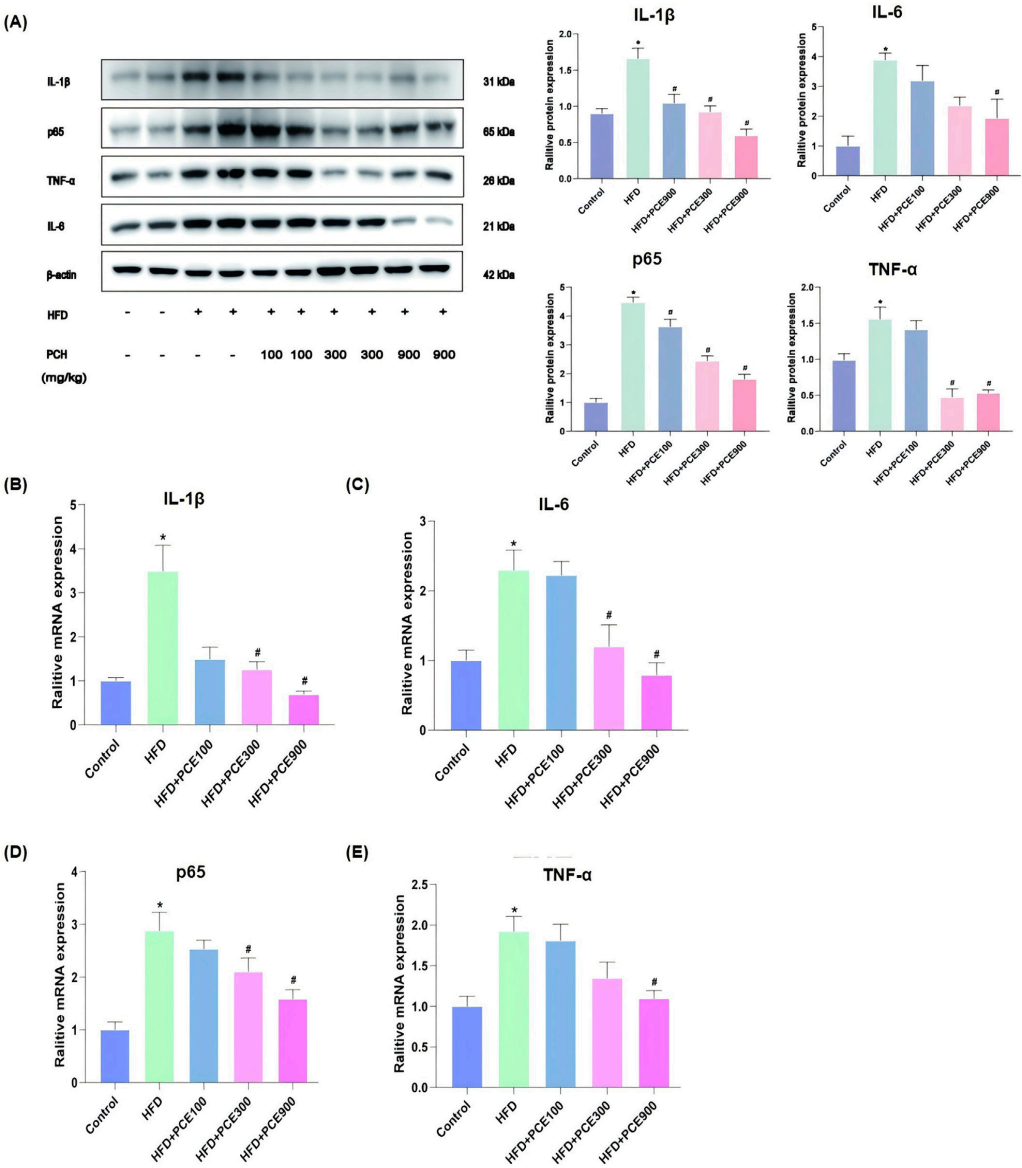
PCE treatment decreased HFD-induced high expression of inflammatory factors. **(A)** Representative Western bolt results of IL-1β, p65, TNF-α and IL-6 and quantification results. **(B)** mRNA relative expression level of IL-1β; **(C)** mRNA relative expression level of IL-6; **(D)** mRNA relative expression level of p65; **(E)** mRNA relative expression level of TNF-α. Data are presented as mean ± SEM (n = 6). ^*^ *p* < 0.05 compared to Control; ^#^ *p* < 0.05 compared to HFD group.

NASH is an inflammatory subtype of NAFLD. Besides fat accumulation, another significant feature of NASH is inflammation. Anti-inflammatory is adopt as therapy for NASH (Xu et al., 2022). Besides energy-sensing, the vital role of AMPK/SIRT1 in inflammation is emerging (Saravia et al., 2020). In view of the activation effect of PCE on AMPK/SIRT1 and to full evaluated the effect of PCE in alleviate NASH, the levels of interleukin-1β (IL-1β), IL-6, p65 and tumor necrosis factor-α (TNF-α) in mice liver tissue were tested by Western blot and qPCR. The representative images of Western blot are shown in Figure 5A. The findings indicate that PCE reduced HFD-induced overexpression of these proteins. Consistently, as show in Figures 5B–E, HFD increased mRNA levels of these four inflammatory factors obviously, and which were reversed by PCE treatment.

### PCE attenuated HFD-induced DEGs and the related canonical pathways and upstream regulators were revealed

Given the diversity of metabolites in PCE, we hypothesize that the mechanisms by which PCE alleviates NASH are diverse. Therefore to fully elucidate the protective mechanism of PCE against HFD-induced NASH, we performed RNA-seq analysis. Under criteria of *p* < 0.05, HFD produced 232 (184 up, 48 down) differentially expressed genes (DEGs) as compared to control group (Supplementary Figure S2), which were attenuated or abolished following PCE treatment (PCE100, 275 up and 38 down; PCE300, 201 up and 21 down; PCE900, 172 up and 143 down). When the data were filtered by HFD, it is apparent that PCE treatment attenuated or abolished HFD-induced aberrant gene expressions (Supplementary Figure S3).

**FIGURE 6:**
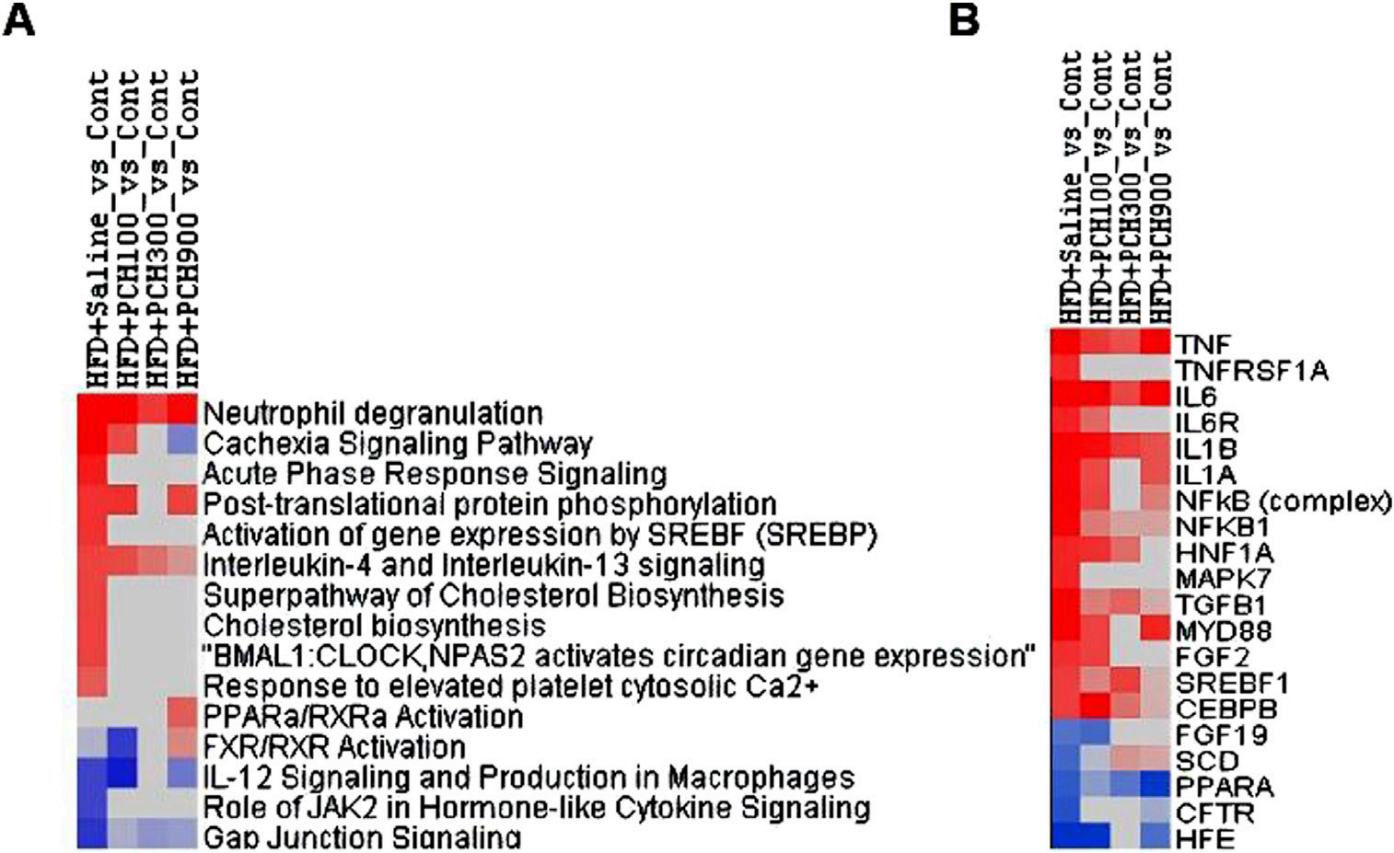
Differentially expressed genes were conducted Ingenuity Pathway Analysis. **(A)**. Top Canonical pathways; **(B)**. Top Upstream regulators.

All DEGs (*p* < 0.05, HFD_vs._Cont, 232; HFD + PCE100_vs._Cont, 313; HFD + PCE300_vs._Cont, 222; HFD + PCE900_vs._Cont, 317) were loaded into Ingenuity Pathway Analysis server for core analysis, followed by comparative analysis. The 15selected canonical pathways is listed in Figure 6A. HFD increased “Neutrophil degradation, cachexia signaling pathway, acute phase response signaling, post-translational protein phosphorylation, activation of SREBP, IL-4 and IL-13 signaling, cholesterol biosynthesis, response to elevated Ca2+, circadian gene expression”, etc. and PCE treatment attenuated or abolished such pathways. On the other hand. HFD decreased “FXR/RXR activation, IL-12 signaling, JAK2 signaling and Gap junction signaling” etc., and PCE treatment alleviated these changes.

Figure 6B shows the 20 selected upstream regulators. HFD increased “TNF, TNFRSF1A, IL6, IL6R, IL1B, IL1A, NFκB, NFκB1”, confirming the findings above, and PCE treatment attenuated or abolished these upregulations. In addition, HFD also increased “HNF1A, MAPK7, TGFB1, MTD88, FGF2, SREBP1, CEBPB” corresponding to HFD-induced NAFLD phenotype, which was ameliorated by PCE treatment. On the other hand, HFD decreased “FGF19, SCD, CFTR, and HFE” which was reversed by PCE to various extent.

## Discussion

This study demonstrated the protective effect against NASH in mice of PCE. Through UPLC-MS/MS, 211 metabolites were identified in PCE and 9 of which were reported to exhibit lipid-lowering effect. After searching literature, we found that the lipid-lowering effects of baicalin (Yang H. et al., 2019; Xiang J. et al., 2019), quercetin (Zhang et al., 2019), atractylenolide (Li, et al., 2022), isorhamnetin (Huang et al., 2016), rhapontin (Wei et al., 2017) and ecsulin (Cheng et al., 2024) are related to the activating of AMPK/SIRT signaling pathway, which provides a basis for PCE to alleviate NASH by activating AMPK/SIRT1 pathway.

C57BL/6J mice underwent significant changes in their general health and biochemical indices following 12-week intake of high-fat chow. These changes included the increased liver indices, mild increases in serum AST and ALT activities, and elevated serum and liver TG levels, in agreement of the literature (Li YJ et al., 2023; Nie et al., 2023; Wang et al., 2020), which demonstrates the effective modeling of NAFLD. PCE effectively inhibited NAFLD development and improved liver function biochemical indices, demonstrating a clear dose-effect relationship.

SIRT1 activates AMPK through deacetylation and AMPK concurrently boosts SIRT1 activity by elevating intracellular NAD^+^ levels (Cantó et al., 2009; Chen et al., 2019; Day et al., 2017). The interaction between SIRT1 and AMPK is essential for regulating molecules related to lipid metabolism and inflammation, significantly influencing NAFLD progression. SREBP-1 is the key transcriptional factors for genes related with the *de novo* lipogenesis pathway. PPAR-α is another AMPK downstream target and is crucial for hepatic lipid oxidation and export (Silva and Peixoto, 2018), its activation leads to the improvement of liver steatosis, inflammation and fibrosis in rodent model (Staels et al., 2013). In current study, PCE elevated the expression levels of AMPK, SIRT1 and PPAR-α, while decreasing SREBP1 levels. Therefore, activating of AMPK/SIRT1 pathway by PCE intake, on the one hand reduced *de novo* synthesis of fat through SREBP1 inhibition, and on the other hand increased fatty acid oxidation through upregulation of PPAR-α activity, and finally improved hepatosteatosis.

Inflammation, closely linked to high-fat diet-induced liver injury and steatohepatitis, is partly driven by the activation of the NF-κB pathway (Ding et al., 2017). SIRT1 deficiency triggers the activation of the NF-κB pathway, leading to increased expression of inflammatory factors like IL-1β, IL-6, and TNF-α, accelerating the progression of NAFLD from simple steatosis to steatohepatitis (Schug et al., 2010). SIRT1 agonists have been shown to exert anti-inflammatory effects by reducing the transcriptional activity of NF-κB p65. Our study demonstrated that PCE attenuated HFD-induced increases in IL-1β, IL-6, TNF-α, and NF-κB p65 at both mRNA and protein levels accompanied by upregulated SIRT1 expression, suggesting its anti-inflammatory effects may be related to SIRT1 activation.

The above two categories, lipid metabolism and inflammation were further supported by IPA analysis of DEGs from RNA-seq. Notably, HFD increased “Neutrophil degradation”, “Cachexia Signaling Pathways” and “Acute Phase Response Signaling” pathways, along with increased upstream markers “TNF, TNFRSF1A, IL6, IL6R, IL1B, IL1A, NFκB, and NFκB1”. This aligns with previous findings (Lian et al., 2020), indicating that anti-inflammation is a key mechanism through which PCE exerts its beneficial effects.

Similarly, HFD increased “post-translational protein phosphorylation” “Cholesterol biosynthesis” “Response to elevated Ca2^+^“. HFD also increased “HNF1A, MAPK7, TGFB1, MTD88, FGF2, CEBPB”, which were ameliorated by PCE treatment. On the other hand, HFD decreased “FXR/RXR activation” and “FGF19, SCD, CFTR, and HFE”, which was reversed by PCE. Most of these molecules are targets for NAHLD (Parlati et al., 2021), supporting the beneficial effects of PCE in lipid metabolism.

PCE treatment also produced other beneficial effects, such as genes for maintaining circadian rhythm, and genes for TGF-β1 signaling, etc., which warrants further investigation.

## Conclusion

In conclusion, the current study demonstrates the effects of PCE in alleviating NASH at least in part by activating the AMPK/SIRT1 pathway.

## Data Availability

The original contributions presented in the study are publicly available. This data can be found here: https://doi.org/10.5281/zenodo.14371978, version 1.
