# Supplementary material for: Ethanol extract of Polygonatum cyrtonema Hua mitigates non-alcoholic steatohepatitis in mice
Source: Front Pharmacol. 2025 Jan 30;15:1487738. doi: 10.3389/fphar.2024.1487738 (PMC11821971; doi:10.3389/fphar.2024.1487738)
Supplement: Supplementary file 1 [file DataSheet1.doc]

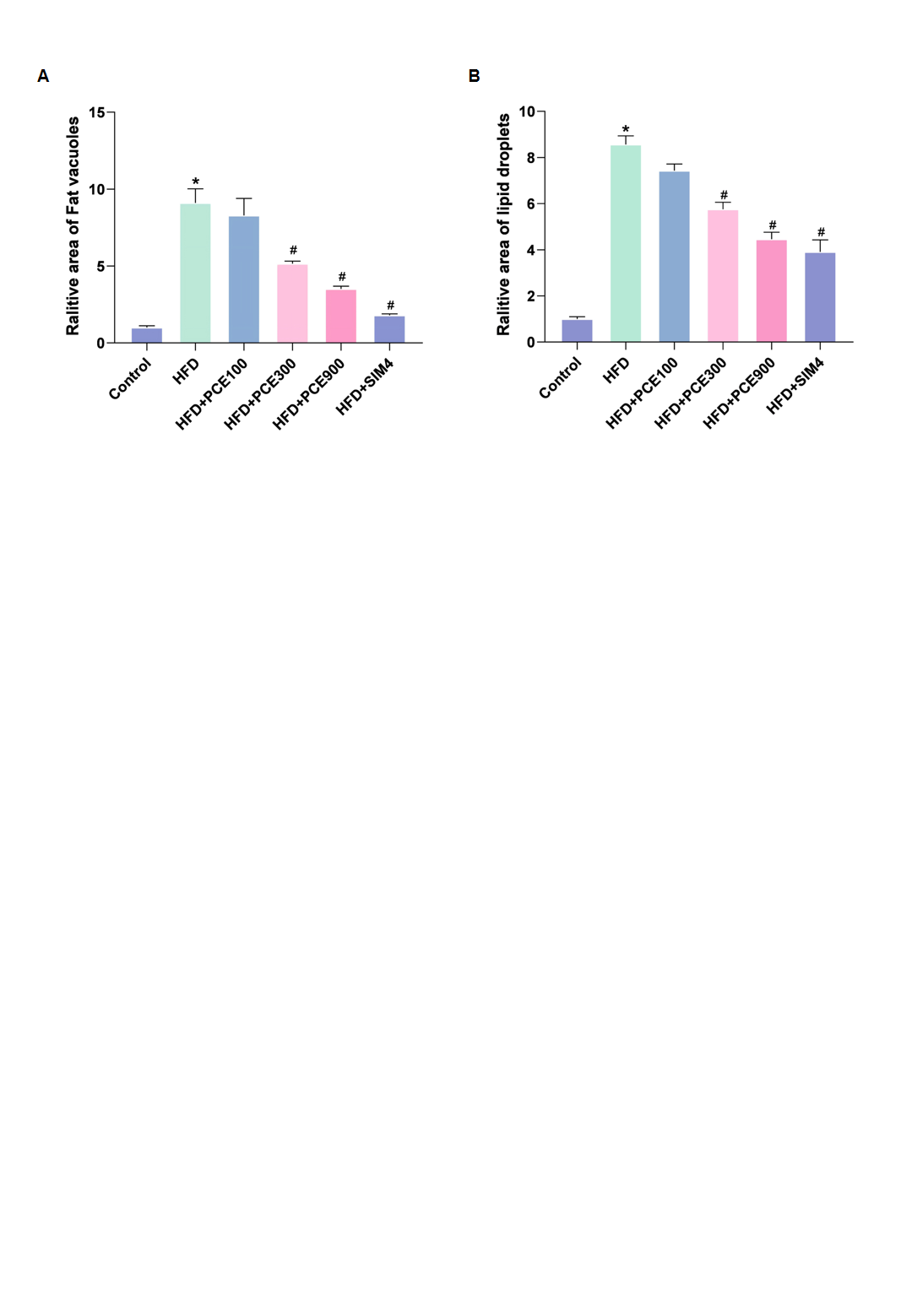
Fig.S1. PCE decreased HFD-induced lipid accumulation in mice liver tissues. A. Relative area (%) of fat vacuoles in H&E staining. B. Relative areas (%) lipid droplets in Oil red O staining. Data are mean ± SD (n = 6). *P < 0.05. *P < 0.05 compared to Control; #P < 0.05 compared to Model.
