# Supplementary figures and images for: Ethanol extract of Polygonatum cyrtonema Hua mitigates non-alcoholic steatohepatitis in mice
Source: Front Pharmacol. 2025 Jan 30;15:1487738. doi: 10.3389/fphar.2024.1487738 (PMC11821971; doi:10.3389/fphar.2024.1487738)

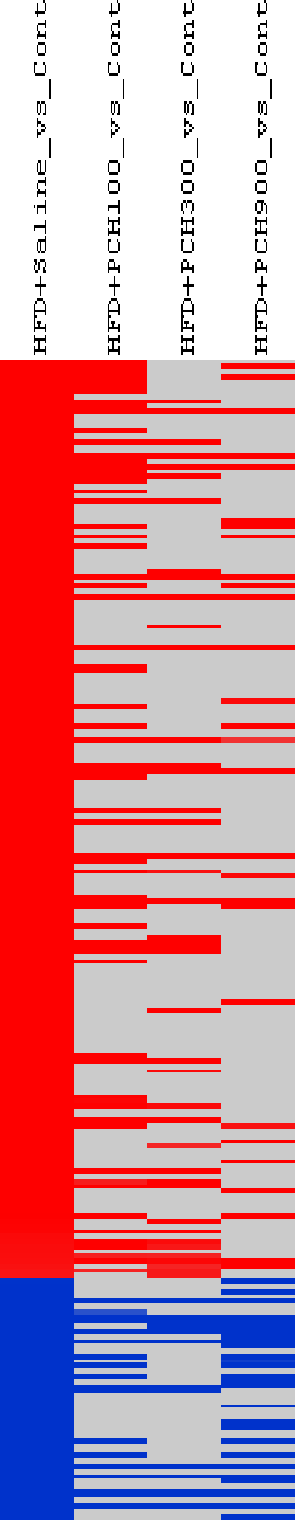

Supplement: Supplementary file 2 [file Image2.tif]

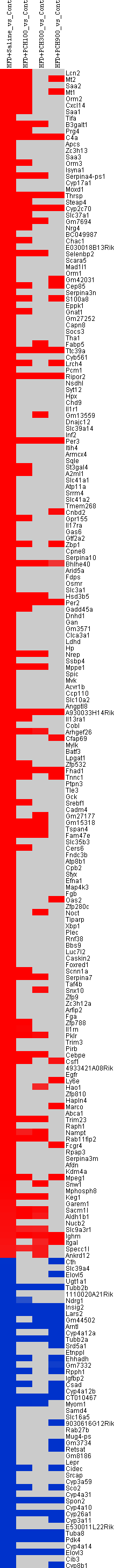

Supplement: Supplementary file 3 [file Image1.tif]
